# MDM4 (MDMX) and its Transcript Variants

**DOI:** 10.2174/138920209787581280

**Published:** 2009-03

**Authors:** F Mancini, G. Di Conza, F Moretti

**Affiliations:** 1 National Council of Research, Institute of Neurobiology and Molecular Medicine, Roma; 2 Inst. of Pathology, Catholic University, Roma; 3 Dept. of Clinical & Experimental Medicine and Pharmacology, University of Messina, Messina, Italy

**Keywords:** MDM4, p53, MDM4-211, MDM4-S, transcript variants, MDM2.

## Abstract

MDM family proteins are crucial regulators of the oncosuppressor p53. Alterations of their gene status, mainly amplification events, have been frequently observed in human tumors.

MDM4 is one of the two members of the MDM family. The human gene is located on chromosome 1 at q32-33 and codes for a protein of 490aa. In analogy to MDM2, besides the full-length mRNA several transcript variants of MDM4 have been identified. Almost all variants thus far described derive from a splicing process, both through canonical and aberrant splicing events. Some of these variants are expressed in normal tissues, others have been observed only in tumor samples. The presence of these variants may be considered a fine tuning of the function of the full-length protein, especially in normal cells. In tumor cells, some variants show oncogenic properties.

This review summarizes all the different MDM4 splicing forms thus far described and their role in the regulation of the wild type protein function in normal and tumor cells. In addition, a description of the full-length protein structure with all known interacting proteins thus far identified and a comparison of the MDM4 variant structure with that of full-length protein are presented. Finally, a parallel between MDM4 and MDM2 variants is discussed.

## FULL-LENGTH MDM4

### Protein Domains

The murine Mdm4 protein (transformed mouse 3T3 cell double minute 4), formerly named Mdmx, has been identified in 1996 as a p53 binding protein [1]. It shows significant homology with the Mdm2 protein (transformed mouse 3T3 cell double minute 2), the best known negative regulator of p53 (Trp53, transformation related protein 53), particularly in the N-terminal p53-binding domain and in the C-terminus of the protein [1]. The human homolog MDM4, (Mdm4 p53 binding protein homolog) has been localized on chromosome 1q32 [2]. Northern blot analysis has revealed a 10-kb human *MDM4* transcript expressed in almost all tissues although with different proportions [2]. The mRNA comprises 11 exons, with the first AUG codon in the second exon. Despite the presence of additional in-frame AUG codons at positions 46, 53 and 61 (starting from the first AUG), there is no evidence of alternative transcripts originating from these start sites [2]. The *MDM4* cDNA codes for a protein of 490 amino acids (aa) (489 in the mouse), with the exon 11 coding for the C-terminal last 189 aa. The structure of the protein is depicted in Fig. (**1**).

It comprises a p53 (human TP53, tumor protein 53) binding domain (BD) between amino acids 24 and 108 according to the NMR study of the analogous MDM2 (Mdm2 p53 binding protein homolog) region [3]. The residues of MDM2 involved in p53 binding [4] are totally conserved in MDM4 (G58, D68, V75, C77 corresponding to MDM4 G57, D67, V74, C76) and mutation of the first of them abolishes p53 binding to MDM4 [5] suggesting high similarity in the association of MDM2 and MDM4 to p53 [6]. However, the crystal structure of the p53-binding domain of MDM4 in complex with a peptide derived from the p53 N-terminus (aa 17-37), has revealed differences between MDM4 and MDM2 binding to p53 [7,8]. Particularly, the hydrophobic cleft on which the transactivation domain of p53 binds is smaller in MDM4 than in MDM2. Coarse graining simulations have confirmed that recognition of p53 is favored in MDM2 with  respect to MDM4 [9]. Further, the N-terminal lid of MDM2 (aa 16-24) that competes for p53 binding under some circumstances, is not conserved in MDM4 [3]. As a further confirmation of the divergence existing between the p53 binding domains of MDM4 and MDM2, the drug-like small molecule Nutlin-3a disrupts the MDM4-p53 complex at a concentration much higher than that required to disrupt MDM2-p53 [8, 10-13].

The other region with high similarity between MDM4 and MDM2 is the C-terminus containing the RING finger motif (aa 437-477). Whereas this motif mediates ubiquitin ligase activity in MDM2, [14, 15] it doesn’t confer the same function to MDM4 *in vivo* [16]. Nevertheless, various groups have shown the ability of MDM4 to potentiate E3 ubiquitin ligase activity of MDM2 and/or to restore function of inactive mutant MDM2 suggesting that MDM4 can contribute to the MDM2 E3 function [17-21]. In addition, the RING finger mediates MDM4-MDM2 association [22-24] although the extreme C-terminus of both proteins is necessary too for their interaction [20, 25]. The residues involved in MDM2-MDM4 interaction have been recognized in some hydrophobic residues (of the RING finger of MDM2) [21]. In addition, mutation of the amino acids disrupting the RING finger structure abolishes this association as well [22, 24].

Within the RING finger a potential nuclear localization signal has been mapped (aa 465-469) composed of a stretch of basic amino acids (RRLKK) [1]. This site has been suggested to mediate nuclear translocation of MDM4 after DNA damage. Indeed, point mutation of a lysine in this stretch abolishes nuclear translocation of MDM4 and its binding to the cytoplasmic receptor importin α [26].

Another recognizable region in MDM4 is the Zinc finger motif between aa 300 and 328. This is a C4 Zinc finger motif of the RanBP2-type Zinc finger family. Particularly, it can be assigned to the related polypeptide family termed Npl4 Zinc finger (NZF), characterized by the presence of only two residues between the first two cysteines [27]. The RanBP2/ NZF fingers have been described as ubiquitin recognition motifs, but the lack in MDM4 of the T_13_F_14_/M_25_ residues would exclude this function [28] leaving unknown the function of this domain.

Close to the Zinc finger motif, it is present a tetrapeptide that represents a canonical caspase cleavage signal (aa 357-361, DVPD) [29]. Indeed, this site is sensitive to caspase activity. The caspase cleavage of MDM4 at this site has been shown to affect protein stability [29].

The central region of MDM4 is rich in acidic residues and for this reason is indicated as acidic domain [16]. So far no specific activities have been attributed to this portion of the protein.

Finally, some residues of MDM4 have been recognized as specific targets of post translational modification:

- K254 and K379 have been identified as SUMO-1 targets. These modifications do not alter MDM4 stability and subcellular localization nor its inhibitory activity toward p53 transcriptional function, leaving unknown the role of these sumoylations [30];
- S96 is phosphorylated by cyclin-dependent kinase 1 (CDK1/Cdc2^p34^). The mutation S96A redistributes MDM4 in the nucleus suggesting that the phosphorylation of this site may affect MDM4 subcellular localization [31];
- S289 is phosphorylated by Casein Kinase 1α (CK1α) This modification strengthens the association between MDM4 and p53 and the inhibition of p53 transcriptional function by MDM4 [32];
- S342 is phosphorylated by Checkpoint Kinases 1 and 2 (Chk1/Chk2) [33, 34]. This modification, as that of the S367 site (see below), is present in U2OS cell line under normal growth conditions but is highly increased after IR [33]. The phosphorylation of this site mediates enhanced degradation of MDM4 [33-35]. Comparative *in vitro* assays indicate that Chk1 is more efficient than Chk2 in inducing this phosphorylation although *in vivo* the knockout (KO) of Chk2 abolishes the DNA-damage induced Ser342^P^ [33];
- S367 is phosphorylated by Chk1/Chk2/Akt [33, 34, 36, 37]. As for S342, the phosphorylation at this MDM4 site is detectable also under normal growth conditions although DNA damage strongly induces it [33, 34, 36, 38]. Phosphorylation at this site regulates MDM4 stability [33-35, 37, 38] and induces binding of different 14-3-3 isoforms to MDM4 (See the following 14-3-3 paragraph);
- S403 is phosphorylated by ataxia telangiectasia mutated (ATM) [35]. It is induced by DNA damage and the S403A MDM4 mutant is more stable than the wild-type (wt) protein indicating also for this phosphorylation a role in MDM4 degradation [35].

## MDM4 INTERACTIONS

### P53 Family Members

MDM4 has been identified by Jochemsen’s group as a p53 binding protein [1]. P53 is one of the main human oncosuppressor genes and is involved in a plethora of cell functions. The crucial role of MDM4 in the regulation of p53 has been assessed by the KO Mdm4 mice. *Mdm4^-/-^* mice die in utero but their death is rescued by concomitant deletion of the *Trp53* gene [39-41]. At the molecular levels, the different activities exerted by MDM4 and MDM2 toward p53 have led to the proposal of three different models of inhibition of p53 function, still debated in the scientific community [42].

In addition to p53, the BD of MDM4 is probably responsible for the association of MDM4 to the other p53 family member, p73α although this has not been experimentally proven [43,44]. On the contrary, *in vivo* binding of MDM4 to p63α and p63γ both the TA and DN variants, have been excluded by two groups suggesting the existence of differences among the p53 family members [44,45]. The activity of MDM4 toward p73α confirms its transcriptional inhibitory function [44].

## MDM2

The binding between MDM4 and MDM2 leads to the formation of a heterodimer, which possesses higher stability in comparison to the homodimer of each protein [18, 21, 23]. In MCF7, majority of MDM4 is in complex with MDM2 suggesting that the heterodimer is the predominant form into the cell [24]. However, the function of the heterodimer has not been completely clarified. Indeed, whereas a growing body of evidence suggests a cooperation of MDM4 in the E3 ligase activity of MDM2 [17-21, 24], a recent report has evidenced the lower stability of a temperature-sensitive mutant p53 (p53A135V) protein in mouse embryo fibroblasts derived from *Mdm4^-/-^* mice [46] suggesting the existence of an antagonizing function of endogenous MDM4 toward the degradative activity of MDM2, at least under some conditions. In addition, it has been reported an antagonizing function of MDM4 toward MDM2-degradative activity when the levels of overexpressed MDM4 exceed those of MDM2 [16].

### 14-3-3 Proteins

The interaction of MDM4 with 14-3-3 family has emerged in recent years. Although a detailed comparison of the affinity of MDM4 to the seven 14-3-3 members has not been carried out, the 14-3-3σ form seems the less efficient in this association [26, 36-38]. The MDM4 region involved in such interaction has been recognized in the peptide 363-369 of MDM4. Particularly, the amino acid S367 is the phosphorylable key residue responsible of this association [26, 34, 36-38]. In addition, the region surrounding residue S342 affects the binding between MDM4 and 14-3-3, at least under normal growth conditions [34]. The outcome resulting from MDM4/14-3-3 association is really puzzling: the major and more detailed effect is the nuclear degradation of MDM4 [26, 34, 38], however stabilization [37] as well as increased cytoplasmic localization of MDM4 [36] have been reported too. Whether these different results rely on the S367-phosphorylation accompanying modifications, or/and on the type of 14-3-3 isoform bound to MDM4, and/or on the DNA damage conditions, it remains to be ascertained.

### Other Proteins

#### CK1α (Casein Kinase 1α)

MDM4 associates to CK1α through a large region encompassing the acidic and the Zinc finger regions and this association has been proven necessary to the MDM4-mediated inhibition of p53 [32].

#### CDK1/Cdc2^p34^ (Cyclin-Dependent Kinase 1)

The association of murine MDM4 to the endogenous Cdc2 has been shown by means of the overexpression of MDM4. The region responsible for the association has not been mapped. It has been hypothesized that this association mediates phosphorylation of MDM4 on aa S96 that in turn affects MDM4 subcellular localization [31].

#### E2F1

The interaction of MDM4 with this factor has been assessed by GST*-*pull down assay and yeast two hybrid screen [47]. These studies have indicated the MDM4 region next to the C-terminus of the BD important for this association [47]. Nevertheless, this association has not been confirmed by *in vivo* coimmunoprecipitation experiments [48]. A reduction of MDM4 protein levels has been reported following overexpression of E2F1 [49] although the molecular mechanisms underlying this reduction remain to be clarified as well as the biological significance of it. Conversely, the ability of MDM4 to repress E2F1 transactivation has been reported [48]. Whether the E2F1-MDM4 association is responsible for any of these effects has not been tested.

#### Hausp/USP7 (Ubiquitin Specific Peptidase 7)

A direct binding of the deubiquitinating enzyme Hausp with MDM4 has been described by Jochemsen’s group [50]. The behavior of MDM4 deletion mutants has indicated that the p53 binding domain of MDM4 is sufficient to coimmunoprecipitate Hausp. However, a MDM4 mutant lacking this domain is still able to bind Hausp with efficiency similar to the wt-protein suggesting that another not well defined region of MDM4 may mediate Hausp binding. The binding of Hausp to MDM4 contributes to stabilize MDM4 protein levels, by counteracting its MDM2-mediated degradation. The current model suggests that in normal growing cells MDM4 levels are controlled by a balanced association with Hausp and MDM2. Under DNA damage, Hausp association to MDM4 is attenuated in an ATM dependent manner leading to a decrease of MDM4 protein levels [50,51].

#### Importin α

Within the RING finger, MDM4 possesses a stretch of basic aa that represents a putative nuclear localization signal [1]. Indeed, upon DNA damage MDM4 translocates in the nucleus, also independently of its shuttling protein MDM2 [52]. GST-pull down experiments have shown interaction of MDM4 with the cytoplasmic receptors importin α [26]. Point mutation of MDM4 basic amino acid, K468, impairs MDM4 binding to importin α and nuclear translocation after DNA damage suggesting a potential role for this association at least under some conditions.

#### P14^Arf^

The binding of MDM4 to p14^ARF^ is controversial since different groups have published opposing results with overexpressed tagged proteins [53-55]. Definitely, it requires confirmation with the endogenous proteins. However, interference of MDM4 towards p14^ARF^ activity [55] as well as of p14^ARF^ towards MDM4 function [54] has been reported suggesting the potential existence of a crosstalk between these two proteins.

#### P21

The binding of MDM4 to the cyclin-dependent kinase inhibitor, p21 (CDKN1A), requires at least three regions, one comprising the BD, the second the central region (aa 233-322), the third encompassing the RING finger domain (aa 423-490) [56]. Whether the three regions represent all contact points or are necessary for the proper conformation of the binding pocket remains to be ascertained. The MDM4 function toward p21 promotes proteosomal turnover of p21.

#### P300

The association between MDM4 and the co-activator p300 has been assessed only by *in vitro* experiments [57]. However, the ability of MDM4 to interfere with p300 activity has been reported. This interference results in decrease of p300-mediated acetylated p53 [58] and in the impairment of the interaction of p300 with the transcriptional factors Smad3 and 4 that in turn invalidates the activity of these last [57].

#### pRB (Retinoblastoma Protein)

Coimmunoprecipitation experiments in tumor cell lines have shown the association between endogenous proteins MDM4 and pRB [59]. Although the MDM4 region responsible for such binding has not been mapped, the C-terminus (aa 405-490) is not required. Further, the possibility that this association is mediated by other proteins cannot be ruled out. In the report of Kitagawa’s group, MDM4 counteracts MDM2-mediated degradation of pRB, thus positively regulating its protein levels and transcriptional function [59].

#### S2

Recently, MDM4 has been shown to interact with the subunit S2 (Rpn1) of the 26S proteasome [56]. In this report, the BD and the C-terminus regions of MDM4 are both required for the association although the C-terminus loss slightly impairs the binding [56] (Di Conza *et al*., *unpublished data*). This association is presumed to confer to MDM4 the ability to promote proteosomal degradation of the cyclin dependent inhibitor p21.

#### SMAD 3 and 4

Similarly to E2F1, contradictory results have been provided about this association [57, 60]. However, an inhibitory function of MDM4 toward these two transcription factors has been reported in two independent reports.

## MDM4 Variants

Analysis of human tumors has evidenced aberrant expression of MDM4 in different human tumors characterized by wild type *TRP53* supporting the hypothesis of an important role of MDM4 in the control of p53 in human tumorigenesis. The more frequent event observed in human tumors is the amplification of the gene [42]. In addition, the presence of shorter forms of MDM4 besides the full-length protein has emerged. The first observation derived from Jochemsen’s group, which reported the presence by immunoblot (WB) analysis of at least 5 shorter MDM4 forms in a panel of 31 human tumor cell lines [61]. Only one of them, resolving at kDa 30,000, was detected in normal cells, melanocytes, suggesting the occurrence of the other forms specifically in tumor cells. Since then, 7 shorter forms of MDM4 have been characterized. All, but one, derived from alternative splicing (Fig. **2**). These transcript variants are described in more details in the following paragraphs.

## MDM4-S

Mdm4-S was the first transcript variant identified in murine and in human cells [62]. Human MDM4-S is the product of an internal deletion of 68 base pairs, occurring at the level of exon 6. This deletion produces a shift of the reading frame after codon 114 and introduces a new translation stop codon following amino acid 140 (Fig. 2b). The result of this splicing is a truncated protein that encodes for the first 114 aa of full-length (fl) MDM4 (the entire p53 binding domain) with the addition of C-terminal 26 aa residues (13 in the murine protein) [16]. In immunoblot, the MDM4-S size of 27kDa is higher than its predicted molecular mass of 15kDa, suggesting the presence of posttranslational modifications as hypothesized for fl-MDM4. In agreement with its structure, MDM4-S associates with p53. However, its affinity toward p53 is approximately 10 fold higher than that of fl-MDM4 (analyzed by GST pull down) [62, 63]. As a consequence, MDM4-S is a more potent suppressor of p53 transcriptional activity. Analysis of nuclear and cytosolic fractions indicates that endogenous MDM4-S is localized in the nucleus [62, 63]. Whether the nuclear localization of MDM4-S may be responsible, at least in part, for the enhanced inhibition of p53 has not been ascertained.

The MDM4-S-mediated impairment of p53 function has been hypothesized as the basis for its oncogenic potential [62]. In addition, the lack of other portions of fl-MDM4 protein should make MDM4-S insensitive to the regulatory modifications thus far reported, particularly those regarding the destabilization of protein levels. Although comparison of MDM4-S and fl-MDM4 protein stability has not been performed, this property would make MDM4-S even more oncogenic that fl-MDM4. In agreement with the hypothesis of higher oncogenic properties of MDM4-S in comparison to fl-MDM4, analysis of tumor cell lines and samples has evidenced levels of *MDM4-S* transcript exceeding those of the *fl-MDM4*, with a certain frequency [62, 64, 65]. On the contrary, in normal tissues the ratio MDM4-S to fl-MDM4 never exceed 1.1 with majority of values <1 [64, 65]. Interestingly, in growing cells in comparison to serum starved cells *MDM4-S* transcript is increased, confirming its positive role in cell growth [62].

Thus far, analysis of *MDM4-S* mRNA and/or protein has been evaluated in three different groups of tumor samples: in a group of 208 gliomas [66], a group of 66 primary soft tissue sarcomas (STS) [64], and a group of 57 papillary thyroid tumors (PTC) [65]. *MDM4-S* is detectable in 78% of the STS samples and in 91% of PTC while the frequency of its overexpression ranges from 19% to 35% in the two groups. In all three groups, the levels of *MDM4-S do* not correlate with increased levels of fl-*MDM4* mRNA suggesting that the overexpression of MDM4-S is independent of *MDM4* mRNA levels.

The analysis of association of MDM4-S overexpression with tumor properties has evidenced a significant correlation with an unfavorable prognosis of STS patients [64]. Intriguingly, in this report there is no association between MDM4-S overexpression and p53 status, suggesting that MDM4-S might exerts its function towards targets other than wt-p53. It would be interesting to investigate whether it exerts any positive regulation of mutant p53. In analogy to data reported for STS, a significant association of *MDM4-S* overexpression has been reported with high grade glioblastomas [66] supporting the hypothesis that MDM4-S, better than fl-MDM4, might contribute to inactivate p53.

## MDM4-A and MDM4-G

MDM4-A and MDM4-G have been characterized in C33A cells [67]. Although the endogenous protein product of their transcripts has not been evidenced, the analysis of their activity has evidenced some potential interesting properties of MDM4 variants.

MDM4-A lacks most of the acidic region (deletion of aa 225-274) (Fig. **2b**). This loss results in a strong susceptibility of this variant to the MDM2 degradative activity, pointing to an important role of this domain in the stability of fl-MDM4. Although detailed studies are necessary to clarify the mechanism that underlie MDM4-A destabilization, it has been proposed that the acidic domain of MDM4 might mask the acidic domain of MDM2 and inhibit its degradative function especially toward p53, which depends also on the presence of this domain [67]. Thus, this MDM4 variant might possess oncogenic properties by functioning in complex with MDM2 to enhance the degradative function of it.

The MDM4-G variant results from an in-frame deletion of aa 27-124, encompassing the p53-binding domain (Fig. **2b**). Interestingly this variant, which is unable to bind p53, still possesses inhibitory activity toward p53 although less pronounced than that of fl-MDM4. This inhibition has been attributed to the stabilization of MDM2 levels and consequently of its oncogenic properties.

These data highlight the complexity of the function of MDM4 variants that might rely on the alteration of the activity of other oncogenic proteins, as MDM2 and/or MDM4, rather than on intrinsic tumor promoting properties.

## MDM4-211

MDM4-211 is a variant of human MDM4 identified in a thyroid tumor cell line [68]. This form derives from an aberrant splicing event between the canonical donor site in exon 2 and a cryptic acceptor site in exon 11 of *MDM4* fl-mRNA. The resulting protein contains the first 26 amino acids and the last 138 amino acids of fl-MDM4 (Fig. **2b**). For this protein too, as for MDM4-S, the predicted molecular weight does not correspond to the molecular weight observed in SDS-PAGE suggesting the presence of covalent modifications. MDM4-211 does not bind p53 because of the almost complete loss of the p53 binding domain. Indeed, direct activity of MDM4-211 toward the p53 oncosuppressor was not observed. On the contrary, MDM4-211 binds MDM2 through the C-terminus and stabilizes its protein levels by increasing the protein half life. Particularly, MDM4-211 seems to function as an inhibitor of MDM2 degradative function causing stabilization of p53 in addition to MDM2. Interestingly, the resulting p53 is transcriptionally inactive, raising the hypothesis that MDM4-211 may stabilize an inactive complex, MDM2-p53, by binding to it. The oncogenic properties of MDM4-211 might therefore rely on both stabilization of the oncoprotein MDM2 and inactivation of p53. In fact, colony assays have shown transforming potential ability of MDM4-211 both in a p53-null and wild type background supporting the hypothesis that the primary target of MDM4-211 is the oncogenic MDM2 [68].

In human tumors, *MDM4-211* was found in 2 out of 16 non–small-cell lung cancers [68]. In addition, *MDM4-211* was found with a frequency of 18% in a group of 83 papillary thyroid carcinomas (PTC) [65]. No correlation with tumor properties has been reported. In most of the tumors expressing MDM4-211, MDM2 showed enhanced protein levels not associated to gene amplification, confirming the molecular model proposed. Importantly, MDM4-211 was never found in normal tissues, suggesting it derives from an aberrant event occurring only in tumor cells [65, 68]. In analogy to what happens for MDM4-S, the expression of *MDM4-211* does not correlate with the levels of fl-*MDM4* transcript suggesting the independency of this splicing event from the abundance of fl-*MDM4* transcript.

Besides the effect toward MDM2, MDM4-211 maintains the interaction with fl-MDM4. Thus, some of its activities could also rely on the deregulation and/or squelching of other factors from fl-MDM4. Particularly, MDM4-211 maintains the 14-3-3 binding site and might therefore decrease the interaction of MDM4 with these proteins and consequently impair the MDM4 degradation mediated by this association, thus enhancing the oncogenic potential of fl-MDM4.

## MDM4-XALT1 and –XALT2

In 2006, along the analysis of MDM2 alternative transcripts induced by UV irradiation in some human tumor cell lines, Lozano’s group found out two alternative transcripts of *MDM4*, called *XALT1* and *XALT2* [69]. *XALT1* derives from a splicing event between exon 5 and exon 10 of *MDM4* fl-mRNA while *XALT2* derives from a splicing between exon 3 and exon 10 (Fig. **2b**). The predicted protein of *XALT1* transcript possesses only the p53 binding domain. As a consequence, it is presumed to bind p53 and to suppress its transcriptional activity as occurs for the MDMX-S form. Conversely, the predicted protein of *XALT2* transcript lacks the p53 binding domain and retains the COOH-terminal RING finger domain. Thus, this variant may bind fl-MDM4 and/or MDM2 and regulate their function. Interestingly, a transcript analogous to XALT2 has been described for MDM2 under the same conditions.

These forms have been evidenced in different tumor cell lines. Their presence has been supposed to be a further layer of regulation of the p53-MDM2-MDM4 network in response to DNA damage. However, their presence after high doses of UV irradiation, which probably induce an apoptotic response, might also represent a survival response of tumor cells. Indeed, XALT1 might limit p53 activity while XALT2 might stabilize MDM2 protein levels and its antiapoptotic function [70]. The expression of these variants in non tumor cells might help to discriminate their function [69].

### Role of MDM4 Variants in Human Tumorigenesis

The two members of the MDM family, MDM2 and MDM4 are characterized by the presence of a large number of splicing variants. For MDM2, 40 splice variants have been described [71] while six for the younger MDM4 have been identified. It has to be pointed out that for majority of these variants it is presently unknown whether they are translated into protein.

Most of them have been recognized only in tumor samples and/or tumor cell lines. The reasons for a so frequent rearrangement of fl-mRNA splicing have not been provided. Some conserved sequence blocks for the splicing factors SC35 and SRp40 have been recognized in *MDM4* and *MDM2* but their function has not been investigated [69]. Similarly, in *MDM2* some regions have been identified having high sequence homology and repeated occurrence (at least 4 times) [71]. However, the same regions are not recognizable in *MDM4*, thus not supporting a role for them at least in MDM4 splicing.

The recovery of some of the MDM4 variants, as well as of MDM2, in tumor cells and the analysis of their activity suggest that they may possess tumorigenic activity. For MDM2 some studies have indeed confirmed such activity in *in vivo* models [72,73]. For MDM4 similar models have not yet been developed.

The discrimination of the oncogenic activities of these variants represents a difficult task. Indeed, their ability to bind and modify the function of their fl-counterparts, MDM2 and MDM4, intertwine their direct activity towards targets as p53 and affects the functional outcome of their expression. Thus, their activity may reside not only in their intrinsic properties but also in their ability to squelch out binding partners from MDM4/MDM2 and/or sequester the fl-proteins. In addition, the ratio of expression levels of these variants to those of fl-MDM proteins represents an additional variable in the study of their function. Indeed, analysis of tumor samples has shown the absence of any correlation between the levels of MDM4-S or MDM4-211 and fl-MDM4 transcript suggesting independency of their expression.

**Fig. (1).**
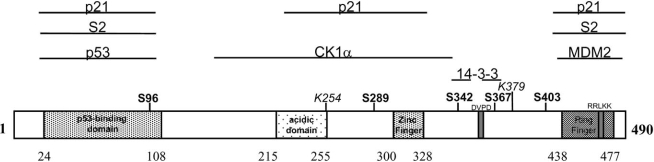
Scheme of MDM4 protein domains and of regions involved in protein-protein interaction. The interaction proteins validated by *in vivo* binding assays are shown. Bold characters represent validated serine phosphorylation sites. Italic characters represent lysine sumoylation sites.

**Fig. (2).**
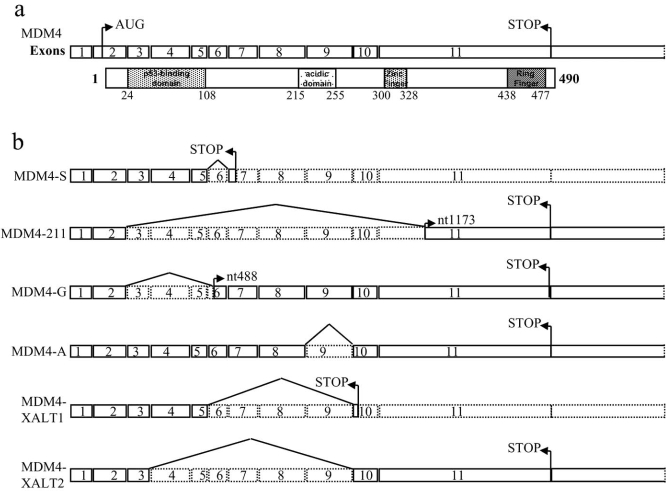
(**a**) Scheme of MDM4 mRNA organization in exons (according to the GeneBank NM 002393) paralleled to the structure of the MDM4 protein regions. (**b**) Scheme of mRNA of MDM4 variants.

Interestingly, the MDM4 variants may be roughly distinguished in two groups: one preserving the p53 binding domain and the other preserving the COOH-terminus where MDM2 binding resides (Fig. **2a** and **b**). With the exception of MDM4-A, the presence of one of these two portions appears to exclude the other. Although, the number of MDM4 variants identified thus far is too low to draw out any certain prediction, this observation might suggest that the central part of MDM4 exerts a regulatory function counterselected by transformation process. In agreement with this hypothesis, most of the MDM4 modifications thus far identified reside in the central part of the protein (Fig. **1**).

Analysis of the association of MDM4 variant expression with tumor properties has evidenced some significant correlations. These data may suggest a potential role of these MDM4 forms as molecular predictors of cancer features.

Finally, an important issue raised by the identification of MDM4 and MDM2 variants is the need to develop accurate detection systems able to distinguish the fl-MDM molecules from their variants in the tumor samples both at the levels of mRNA as well as of protein. This requires the development of specific primers for RT-PCR and most importantly of specific antibodies in immunohistochemistry. This aspect seems of particular relevance in view of the lack of a correlation between the presence of these variants and the levels of the full-length transcript.

## CONCLUSIONS

The discovery of MDM4 in 1996 has added a further layer of complexity to the regulation of p53 by MDM2. Nowadays, the different molecular activities exerted by the two MDM proteins toward p53 have originated at least three models of *in vivo* function, indicating the difficulties to join in an unmatched view all experimental data thus far provided. The uncovering of different variants of both MDM2 and MDM4 increases even more the complexity of this picture that requires additional investigation of MDM function, especially along the tumorigenic process. It represents an interesting challenge in the investigation of tumor biology and of particular relevance in view of the proposal of targeted therapies toward specific molecules, such as those based on the release of p53 oncosuppressive function from MDM constraint.
